# Differences in outcomes of hospitalizations for heart failure after SGLT2 inhibitor treatment: effect modification by atherosclerotic cardiovascular disease

**DOI:** 10.1186/s12933-021-01406-3

**Published:** 2021-10-23

**Authors:** Shih-Chieh Shao, Kai-Cheng Chang, Swu-Jane Lin, Shang-Hung Chang, Ming-Jui Hung, Yuk-Ying Chan, Edward Chia-Cheng Lai

**Affiliations:** 1 Department of Pharmacy, Keelung Chang Gung Memorial Hospital, Keelung, Taiwan; 2 School of Pharmacy, Institute of Clinical Pharmacy and Pharmaceutical Sciences, College of Medicine, National Cheng Kung University, No. 1, University Road, Tainan, 701 Taiwan; 3 Department of Pharmacy, Linkou Chang Gung Memorial Hospital, Taoyuan, Taiwan; 4 Department of Pharmacy Systems, Outcomes and Policy, College of Pharmacy, University of Illinois at Chicago, Chicago, IL USA; 5 Section of Cardiology, Department of Internal Medicine, Linkou Chang Gung Memorial Hospital, Taoyuan, Taiwan; 6 Center for Big Data Analytics and Statistics, Linkou Chang Gung Memorial Hospital, Taoyuan, Taiwan; 7 Chang Gung University, College of Medicine, Taoyuan, Taiwan; 8 Section of Cardiology, Department of Internal Medicine, Keelung Chang Gung Memorial Hospital, Keelung, Taiwan; 9 Department of Pharmaceutical Material Management, Chang Gung Medical Foundation, Taoyuan, Taiwan

**Keywords:** Sodium-glucose cotransporter 2 inhibitors, Atherosclerotic cardiovascular diseases, Hospitalization for heart failure, Effect modification, Anti-diabetic drugs

## Abstract

**Background:**

The treatment effects on hospitalization for heart failure (hHF) from sodium-glucose cotransporter 2 (SGLT2) inhibitors may vary among type 2 diabetes (T2D) patients depending on whether or not they have established atherosclerotic cardiovascular diseases (ASCVD). We aimed to examine differences in hHF outcomes after dapagliflozin or empagliflozin use between T2D patients with and without a history of established ASCVD.

**Methods:**

We conducted a retrospective multi-institutional cohort study in Taiwan. We included T2D patients newly receiving dapagliflozin or empagliflozin during 2016–2019, and followed them up until December 31, 2020. We implemented 1:1 propensity score matching to create homogenous groups for comparisons. We generated Cox proportional hazard models to compare the risk of hHF between dapagliflozin and empagliflozin (reference group). We included interaction terms of SGLT2 inhibitor and ASCVD history in the regression models to examine effect modification by ASCVD.

**Results:**

We included a total cohort of 9,586 dapagliflozin new users and 9,586 matched empagliflozin new users. The overall hHF risks were similar for dapagliflozin and empagliflozin (HR: 0.90, 95% CI 0.74–1.09). However, differential hHF risks between dapagliflozin and empagliflozin were observed only in the subgroup without ASCVD (HR: 0.67, 95% CI 0.49–0.90), while not in the subgroup with ASCVD (HR: 1.12, 95% 0.87–1.45), and the p-value for examining interaction was 0.0097.

**Conclusion:**

In this study, history of established ASCVD was associated with different hHF risks among SGLT2 inhibitors. For T2D patients without ASCVD, dapagliflozin may offer a more favorable hHF reduction effect, compared to empagliflozin, in clinical practice. Future prospective studies should be conducted to validate our findings.

**Supplementary Information:**

The online version contains supplementary material available at 10.1186/s12933-021-01406-3.

## Introduction

Sodium glucose co-transporter 2 (SGLT2) inhibitors, the second-line treatment for type 2 diabetes (T2D) with their pleiotropic metabolic effects [1–5], offer robust benefits with an approximately 30% reduction in risk of hospitalization for heart failure (hHF) in patients with type 2 diabetes (T2D) [6–16]. However, it is noteworthy that some studies have provided evidence implying the magnitude of hHF risk reduction from the treatment with SGLT2 inhibitors may vary between patients with established atherosclerotic cardiovascular disease (ASCVD) and those without. For example, compared to placebo, the DECLARE-TIMI 58 trial has found that dapagliflozin reduces the risk of hHF in T2D patients without established ASCVD by 36%, but only 22% in those with established ASCVD [17]. A recent study by Patorno et al. using real-world data found T2D patients receiving SGLT2 inhibitors had lower risk of hHF, compared to those receiving DPP4 inhibitors, but the magnitudes of the risk differences varied between patients with established ASCVD (44%) and those without established ASCVD (54%) [18]. Similar findings came from another study using Asian population data [19]. However, a major issue with these studies was the lack of statistical examinations to test for effect modification by ASCVD on hHF risk.

Recently, Shao et al. analyzing Taiwan’s multi-institutional electronic medical records (EMR) database found that the use of dapagliflozin may have more favorable outcomes on heart failure, compared to the use of empagliflozin [20]. However, the study only focused on T2D patients without a history of established ASCVD; it was unclear whether SGLT2 inhibitors produced different hHF risk profiles in patients with ASCVD. The probable effect modification of hHF outcomes through history of established ASCVD has not been well evaluated. Therefore, this study aims to compare the risk of hHF outcomes among T2D patients receiving dapagliflozin or empagliflozin. We stratified by patients’ baseline history of ASCVD and examined whether the hHF outcomes were modified by ASCVD history. We hypothesized that the hHF outcomes after SGLT2 inhibitor treatment would differ between T2D patients with established ASCVD and those without. Understanding the association between the history of established ASCVD and outcomes of hHF risk can help clinical physicians to select the most appropriate drug.

## Methods

### Data source

This retrospective cohort study is based on Taiwan’s extensive, multi-institutional Chang Gung Research Database (CGRD). CGRD comprises seven Chang Gung Memorial Hospitals located throughout Northern and Southern Taiwan, and contains the ﻿EMR of 1.3 million patients (6% of Taiwan’s population). Disease identification in the CGRD follows the International Classification of Diseases, Ninth Revision, Clinical Modification (ICD-9-CM) prior to 2016, and ICD-10-CM thereafter. For the purpose of assuring patients’ baseline status in regard to cardio-metabolic and cardiovascular conditions, the database also includes laboratory examination data. Details of data structures and representativeness of the CGRD are described elsewhere [21], and many of the diagnostic codes used in CGRD have been validated [22–24]. The Institutional Review Board of Chang Gung Medical Foundation approved this study under ID# 201801493B0.

### Study population

For the analysis we adopted a new-user design with an active-comparator to minimize potential selection bias and other unmeasured confounding factors [25]. We included adult patients with T2D newly receiving at least two prescriptions of dapagliflozin or empagliflozin from 2016 to 2019. We considered all SGLT2 inhibitor use to be new use since SGLT2 inhibitors were only approved in Taiwan from 2016 onwards. The index date was defined as the first dispensing date of dapagliflozin or empagliflozin. We excluded patients with no clinical visit before the index date to ensure that we had sufficient data for baseline evaluation. We also excluded patients without a laboratory examination, including glycated hemoglobin A1c (HbA1c), estimated glomerular filtration rate (eGFR), systolic blood pressure (SBP) and diastolic blood pressure (DBP) at baseline, to ensure the study patients were under the routine care of the study hospitals. We excluded patients with baseline eGFR less than 30 ml/min/1.73 m^2^ because, following the drug label information, SGLT2 inhibitor use was not suggested for them [26].

### Outcomes definition and follow-up

The study outcome was hHF, as determined by the clinical diagnosis (ICD-10-CM codes: I50) at any point in the hospital discharge records. The diagnosis codes for heart failure in CGRD have been validated by previous study with positive predictive value of 89.8% [20]. We followed up patients from the index date to December 31, 2020, the occurrence of hHF, patients’ death or their last clinical visit based on intention-to-treat method [27]. Additional file 1: Figure S1 presents the details of the study design.

### Covariates

We determined potential confounding covariates on the basis of expert opinion and previous studies [12–14, 28–31]. We retrieved the data from a year prior to the index date for patients’ baseline co-morbidities including the history of established ASCVD (i.e., coronary heart disease, ischemic stroke and periphery artery diseases), cardiovascular diseases (i.e., hypertension, hyperlipidemia, atrial fibrillation and heart failure), diabetic complications (i.e., diabetic nephropathy, neuropathy and retinopathy) and other chronic diseases (i.e., liver disease, chronic obstructive pulmonary disease (COPD), schizophrenia and cancer). Charlson comorbidity index (CCI) was included as the composite score for patients’ disease burdens [32]. We retrieved data of the 3 months prior to the index date for concomitant anti-hyperglycemic agents and other co-medications, including statins, anti-platelets and anti-hypertensives. We also included indicators for diabetes (i.e., HbA1c), renal functions (i.e., eGFR) and some risk factors for heart failure (i.e., SBP and DBP), based on the most recent laboratory and examination data before the index date [33]. Other covariates related to medical care utilization (i.e. hospital levels and specialties of the prescriber) on the index date were also included. The details of co-morbidity and concomitant medications are presented in Additional file 1: Table S1 and Table S2.

### Statistical analyses

To afford more homogeneous comparisons, we used the propensity score method to generate comparable groups [34, 35]. We estimated the propensity scores for the new dapagliflozin or empagliflozin users by using multivariable logistic regression models based on all baseline characteristics listed in Table 1. We implemented a nearest neighbor matching algorithm to minimize distance within matched sets on the propensity score scale with 8 → 1 greedy matching [36]. One user of empagliflozin was selected for each dapagliflozin user, based on propensity score matching. We calculated absolute standardized mean differences (ASMD) to evaluate the differences between the two comparison groups. An ASMD < 0.1 indicates a negligible difference between the two treatment groups [37]. We performed Cox proportional hazards regression modeling after propensity score matching to estimate hazard ratios (HR) and 95% confidence intervals (CI) on the risk of hHF. Empagliflozin users were considered the reference group because empagliflozin was the most frequently prescribed SGLT2 inhibitor in Taiwan during the study period. Specifically, we have included interaction terms in the regression models to examine effect modification by the history of established ASCVD (with vs. without). We considered results with a 2-sided P < 0.05 statistically significant. Statistical analyses were performed using SAS Enterprise Guide (Version 7.1; SAS Institute Inc., Cary, NC, USA).

**Table 1:** Baseline characteristics of propensity score matched cohort

	All patients			Patients with ASCVD			Patients without ASCVD		
	Dapagliflozin (N = 9586)	Empagliflozin (N = 9586)	ASMD	Dapagliflozin (N = 2401)	Empagliflozin (N = 2434)	ASMD	Dapagliflozin (N = 7185)	Empagliflozin (N = 7152)	ASMD
Age, mean (SD) years	59.9 (11.6)	60.0 (12.0)	0.01	63.6 (10.3)	63.8 (10.8)	0.01	58.6 (11.7)	58.7 (12.2)	0.01
Female, %	40.2	40.2	0.01	28.0	27.7	0.05	44.3	44.5	< 0.01
HbA1c, mean (SD) %	8.7 (1.6)	8.7 (1.7)	< 0.01	8.6 (1.6)	8.5 (1.7)	0.04	8.8 (1.6)	8.8 (1.8)	<0.01
SBP, mean (SD) mmHg	140.0 (19.3)	140.0 (19.7)	< 0.01	138.7 (19.9)	137.9 (20.0)	0.04	140.4 (19.1)	140.7 (19.6)	< 0.01
DBP, mean (SD) mmHg	78.7 (12.1)	78.6 (12.0)	< 0.01	77.9 (12.2)	77.1 (12.0)	0.06	79.0 (12.1)	79.2 (12.0)	0.01
eGFR, mean (SD) ml/min/1.73 m^2^	92.8 (27.1)	92.3 (31.6)	0.01	86.3 (25.2)	84.4 (27.9)	0.07	94.9 (27.4)	95.1 (32.6)	< 0.01
Hospital level, %			0.03			< 0.01			0.03
Medical centers	54.4	54.5		55.6	55.9		54.0	54.0	
Regional hospitals	27.2	26.6		29.2	29.1		26.6	25.8	
District hospitals	18.4	18.9		15.3	15.0		19.5	20.2	
Specialty of prescriber, %			0.03			0.02			0.03
Metabolism & Endocrinology	58.0	57.5		32.8	34.1		66.4	65.4	
Cardiology	28.9	28.9		55.8	54.5		19.9	20.2	
Others	13.2	13.6		11.5	11.5		13.8	14.4	
Previous hospitalizations, %	15.3	15.1	< 0.01	28.7	29.8	0.02	10.8	10.1	0.02
ASCVD, %	25.1	25.4	< 0.01	100.0	100.0	–			
Coronary heart disease	21.3	21.7	0.01	85.1	85.6	0.02	–	–	–
Ischemic stroke	3.9	3.3	0.03	15.4	13.2	0.06	–	–	–
Peripheral artery disease	1.3	1.5	0.02	5.0	6.0	0.04	–	–	–
Comorbidity, %									
Hypertension	67.6	68.0	< 0.01	76.5	76.8	< 0.01	64.6	65.0	< 0.01
Hyperlipidemia	72.6	72.3	< 0.01	72.4	71.8	0.01	72.6	72.5	< 0.01
Heart failure	5.7	5.8	< 0.01	14.5	14.6	< 0.01	2.7	2.8	< 0.01
Atrial fibrillation	3.2	3.3	< 0.01	5.4	4.6	0.03	2.5	2.9	0.02
Diabetic retinopathy	8.0	7.8	< 0.01	6.6	6.9	0.01	8.5	8.2	0.01
Diabetic neuropathy	9.0	8.3	0.02	7.6	7.6	< 0.01	9.5	8.5	0.03
Diabetic nephropathy	23.8	23.8	< 0.01	16.7	18.1	0.04	26.2	25.8	0.01
COPD	3.1	3.1	< 0.01	5.3	5.3	< 0.01	2.3	2.4	< 0.01
Liver disease	17.2	17.6	0.01	12.2	12.2	< 0.01	18.8	19.4	0.01
Depression	1.5	1.6	< 0.01	1.3	1.8	0.04	1.6	1.5	< 0.01
Schizophrenia	0.4	0.4	< 0.01	0.3	0.3	< 0.01	0.5	0.5	< 0.01
Cancer	6.0	6.0	< 0.01	5.3	6.2	0.04	6.3	5.9	0.02
CCI, mean (SD) scores	2.5 (1.5)	2.5 (1.5)	0.01	2.8 (1.7)	2.8 (1.7)	0.05	2.4 (1.4)	2.4 (1.4)	< 0.01
Co-medications, %									
Anti-platelet agents	32.8	32.7	< 0.01	77.8	79.6	0.04	17.7	16.8	0.02
Anti-coagulant agents	3.3	3.4	< 0.01	5.3	5.0	0.01	2.6	2.9	0.02
Beta blockers	28.4	28.5	< 0.01	55.3	55.3	< 0.01	19.4	19.3	< 0.01
ACEI / ARB	60.7	61.1	< 0.01	74.6	72.7	0.04	56.1	57.1	0.02
Calcium channel blockers	40.3	41.2	0.02	50.5	45.9	0.09	36.9	39.6	0.06
Spironolactone	2.5	2.3	0.01	4.5	3.8	0.04	1.8	1.8	< 0.01
Other diuretics	9.0	9.6	0.02	13.7	13.7	< 0.01	7.5	8.1	0.03
Digoxin	1.1	1.1	< 0.01	2.1	1.4	0.05	0.8	0.9	0.02
Nitrate	6.3	6.8	0.02	19.6	20.3	0.02	1.9	2.2	0.02
Statin	66.3	66.2	< 0.01	77.0	79.3	0.06	62.7	61.7	0.02
Fibrate	9.1	10.0	0.03	8.1	8.8	0.02	9.4	10.4	0.03
Ezetimibe	11.6	11.8	< 0.01	14.7	15.6	0.03	10.6	10.5	< 0.01
Metformin	87.9	87.7	< 0.01	86.8	86.0	0.02	88.2	88.2	< 0.01
Sulfonylurea	54.8	54.5	< 0.01	55.1	53.1	0.04	54.7	55.0	< 0.01
DPP4 inhibitors	56.6	56.9	< 0.01	56.6	54.5	0.04	56.7	57.7	0.02
Acarbose	16.1	15.7	0.01	18.2	16.1	0.05	15.4	15.5	< 0.01
Glinides	1.8	2.1	0.02	1.4	2.1	0.05	1.9	2.1	0.01
Thiazolidinediones	22.2	21.9	< 0.01	20.6	21.2	0.01	22.7	22.2	0.01
GLP-1 RAs	0.9	0.9	< 0.01	0.8	0.6	0.02	1.0	1.0	< 0.01
Insulins	16.7	16.7	< 0.01	14.0	14.5	0.01	17.6	17.5	< 0.01
NSAID	7.9	8.0	< 0.01	8.3	8.0	0.01	7.8	8.0	< 0.01

ACEI: angiotensin converting enzyme inhibitors; ARB: angiotensin II receptor blockers; ASCVD: atherosclerotic cardiovascular disease; ASMD: absolute standardized mean difference; CCI: Charlson Comorbidity Index; COPD: chronic obstructive pulmonary disease; DBP: diastolic blood pressure; DPP4: dipeptidyl peptidase-4; eGFR: estimated glomerular filtration rate; GLP-1 RAs: glucagon-like peptide-1 receptor agonists; HbA1c: glycated hemoglobin A1c; NSIAD: non-steroidal anti-inflammatory drug; SBP: systolic blood pressure; SD: standard deviation

### Sensitivity analyses

We conducted several sensitivity analyses to examine the robustness of our results. First, to ensure the outcome validity, we re-defined hHF to include only diagnosis from specialized cardiologists at discharge. Second, we used heart failure diagnosis codes at discharge plus the serum B-type Natriuretic Peptide (BNP) levels at two cutoffs of 100 pg/ml and 400 pg/ml during hospitalization to redefine heart failure. BNP levels appear useful for the detection of heart failure, with a cutoff < 100 pg/ml excluding heart failure and > 400 pg/ml a possible diagnosis of heart failure [38]. Third, to reduce the impact of patients’ non-adherence to treatment or loss to follow-up, we performed on-treatment analysis and censored patients exceeding 90 days without a prescription refill, or who switched to another SGLT2 inhibitor or other index medication. Finally, we excluded patients if hHF occurred within 90 days or 180 days after the index date to avoid confounding since hHF that occurs in such a short period of time is likely to be caused by patients’ underlying diseases rather than being the outcome of medications [39].

### E-value analyses

We calculated E-value on the basis of our study outcomes. E-value was developed by Vanderweele et al. to estimate the minimum strength of association that an unmeasured confounder, conditional on the measured covariates, would require to fully explain away the observed associations [40]. For example, E-value 2.0 indicates that an unmeasured confounder associated with both exposure and outcome by a hazard ratio of at least 2 times would eliminate the observed associations.

## Result

### Study population

**Fig. 1:**
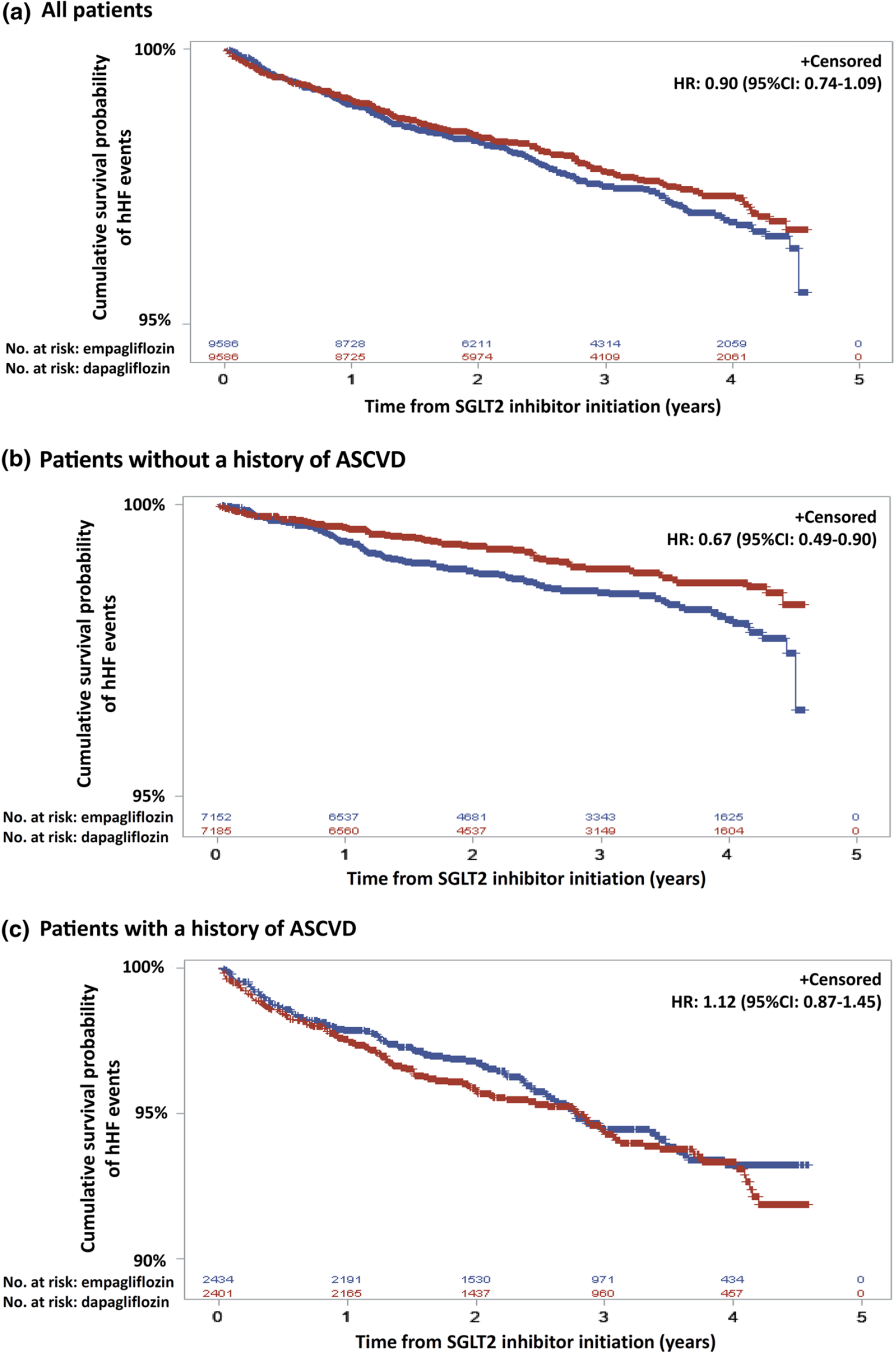
The risk of hospitalization due to heart failure between dapagliflozin new users and empagliflozin new users, matched by propensity score

We identified a total of 10,274 dapagliflozin and 12,030 empagliflozin new users after applying study inclusion and exclusion criteria (Additional file 1: Figure S2). Their baseline characteristics are presented in Additional file 1: Table S3. After 1:1 propensity score matching, we included 9586 dapagliflozin and 9586 matched empagliflozin new users with well-balanced baseline characteristics (all ASMDs < 0.1) (Table 1). For example, for dapagliflozin new users the mean age, HbA1c and eGFR values were 59.9 (SD 11.6) years, 8.7 (SD 1.6) % and 92.8 (SD 27.1) ml/min/1.73 m^2^, respectively. This was similar to empagliflozin new users with a mean age of 60.0 (SD 12.0) years, HbA1c of 8.7 (SD 1.7) % and eGFR of 92.3 (SD 31.6) ml/min/1.73 m^2^, respectively. At baseline, 25.1% of dapagliflozin new users and 25.4% of empagliflozin new users had a history of ASCVD. The mean follow-up periods were similar for dapagliflozin (2.7 years) and empagliflozin (2.7 years) new users. Without stratification by history of ASCVD, there was no significant difference in hHF incidence between dapagliflozin (7.56/1000 person-years) and empagliflozin new users (8.39/1000 person-years) (Additional file 1: Table S4) with an HR of 0.90 (95% CI 0.74–1.09; E-value: 1.46) (Fig. 1a).

### Examination of interaction for history of established ASCVD

Among patients without a history of established ASCVD, and newly receiving dapagliflozin (3.62/1000 person-years), lower hHF risks were observed, compared to those receiving empagliflozin (5.42/1000 person-years), with an HR of 0.67 (95% CI 0.49–0.90; E-value: 2.35) (Fig. 1b). However, among patients with a history of established ASCVD, we found similar hHF risks (HR: 1.12; 95% CI 0.87–1.45; E-value: 1.49) for dapagliflozin users (19.86/1000 person-years) and empagliflozin users (17.62/1000 person-years) (Fig. 1c). Significant effect modification for the association between ASCVD history and hHF risk was found (P value for interaction: 0.0097, Table 2). We present the number of patients at risk, patients with hHF outcomes and patients censored in Additional file 1: Table S5.

**Table 2:** Effect modification on hHF between dapagliflozin and empagliflozin by history of established ASCVD

	Dapagliflozin	Empagliflozin	Dapagliflozin vs. empagliflozin within the strata of ASCVD (+) and ASCVD (−)
ASCVD (+)	1.12 (0.87–1.45)	1 (reference)	1.12 (0.87–1.45)
ASCVD (-)	0.21 (0.15–0.28)	0.31 (0.24–0.40)	0.67 (0.49–0.90)
ASCVD (+) vs. ASCVD (−) within the strata of dapagliflozin and empagliflozin	5.47 (4.08–7.33)	3.24 (2.48–4.23)	

^*^Test for interaction: 0.0097

### Sensitivity analyses

**Fig. 2:**
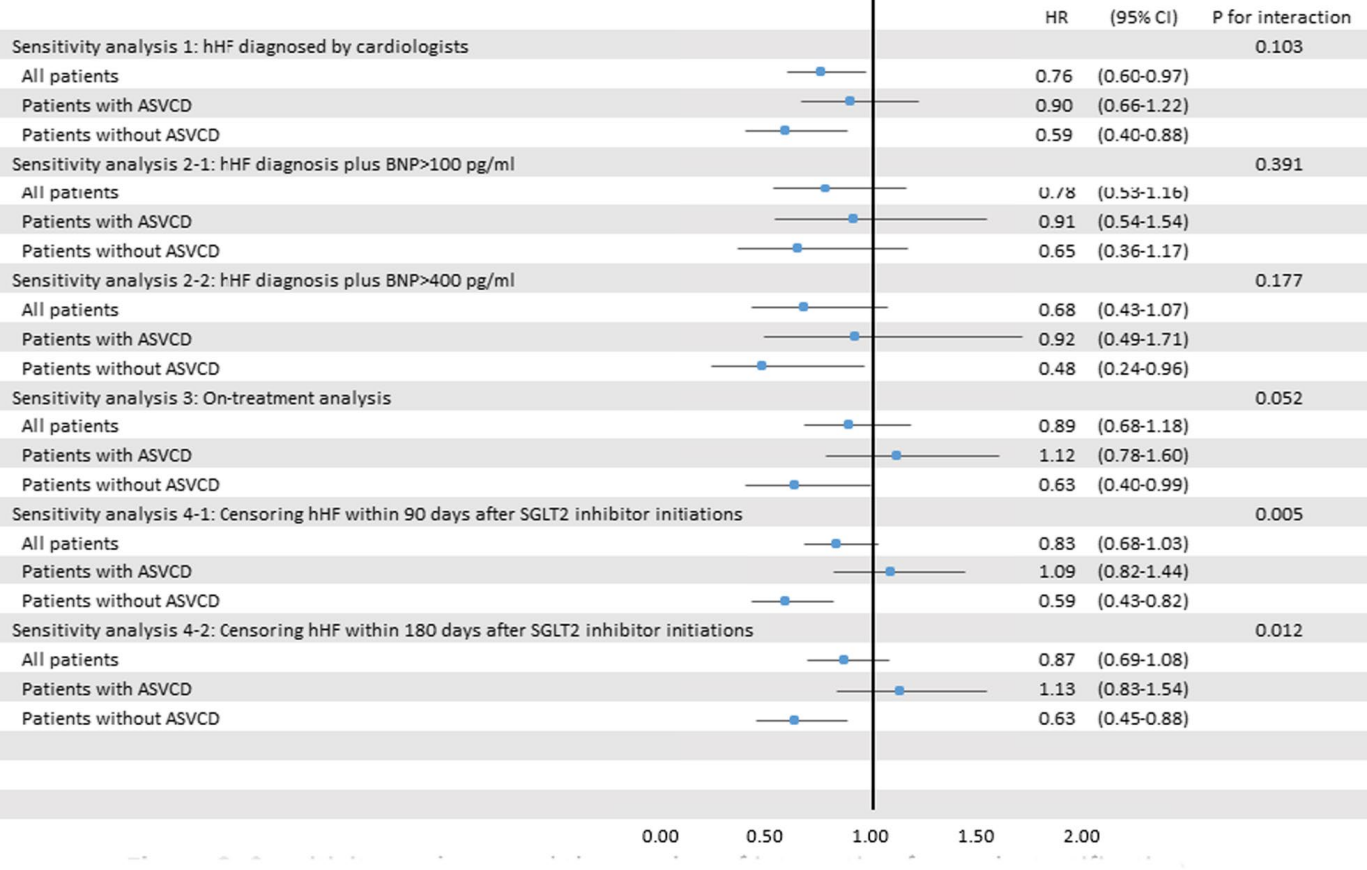
Sensitivity analyses and the p-value of interaction for each stratification

The sensitivity analyses showed the results were robust. Specifically, when we identified hHF using stricter definitions, the results remained consistent with the main analyses. The on-treatment analyses also showed similar results when we stratified patients by their history of established ASCVD (Fig. 2).

## Discussion

The study results indicated the use of dapagliflozin and empagliflozin had a similar effect on hHF outcomes in all T2D patients, of whom approximately 75% were without history of established ASCVD. Notably, when we stratified patients into those with and without a history of established ASCVD, we found the hHF effects of dapagliflozin and empagliflozin to be different. That is, compared to empagliflozin, dapagliflozin was associated with a greater effect of reducing hHF in patients without a history of ASCVD, but this association was not observed in patients with a history of ASCVD. This may reflect the fact that having a history of ASCVD acted as an effect modifier when using SGLT2 inhibitors. These findings highlight the need to consider patients’ history of ASCVD when selecting SGLT2 inhibitors.

RCTs and real-world cohort studies have demonstrated that the use of SGLT2 inhibitors reduces the incidence of hHF in T2D patients. Specifically, the DECLARE-TIMI 58 trial found that dapagliflozin offers more hHF risk reduction in T2D patients without established ASCVD, compared to those with established ASCVD [17]. In addition, in a previous real-world study which focused on T2D patients without established ASCVD, dapagliflozin was proven to offer more reduction of heart failure risk than empagliflozin [20]. Our study further evaluated possible effect modifications among different SGLT2 inhibitors, based on the patients’ baseline history of ASCVD. We found the use of dapagliflozin was associated with a lower risk of hHF in patients without established ASCVD, while this association was not observed in patients with established ASCVD. A plausible explanation may be found in the different pharmacokinetics and differences in SGLT2 and SGLT1 receptor selectivity. A recent study by Lin et al. found that compared to empagliflozin, dapagliflozin may have better glucose-lowering effects, and also lower the risk of renal function decline in T2D patients in clinical practice [41], and that improvement in these conditions was associated with lower risk of hHF. Several studies have also indicated that dapagliflozin, having longer pharmacological effects, compared to empagliflozin, may lead to less variability in SBP and glycemic levels, and thus may be associated with reduced risk of heart failure [42–47]. In particular, recent meta-regression analyses have indicated better risk reduction for hHF events with decreasing SGLT2 receptor selectivity [48]. The SGLT2:SGLT1 receptor selectivity ratio of dapagliflozin is 1200:1, lower than that of empagliflozin (2500:1) [49], and this relatively higher selectivity of SGLT1 receptors in the case of dapagliflozin may decrease variations in postprandial blood glucose levels, since SGLT1 receptors can be found predominantly in the human intestine. This may also result in additional benefits with regard to heart failure risk reduction [50–52]. Recent studies have reported that inhibition of SGLT1 receptors in the heart may decrease hyperglycemia-induced generation of reactive oxygen species, leading to potential prevention of heart failure [53, 54]. In addition, compared to empagliflozin, dapagliflozin did not increase plasma aldosterone and noradrenaline levels, which may also prove beneficial for the prevention of hHF [55].

In this study, we used standard statistical approaches to confirm effect modification through the history of established ASCVD. It is noteworthy that the superior effects of dapagliflozin over empagliflozin as regards the risk of hHF were only observed in patients without a history of established ASCVD. The mechanisms behind this effect modification remain unclear. One possible explanation is that patients with established ASCVD may develop ischemic cardiomyopathy associated with lower cardiac functions [56], since the diuretic and natriuretic effects of the SGLT2 inhibitor that improve preload and afterload have become relatively restricted. That is, the history of established ASCVD may be a crucial predisposing factor leading to hHF. This may also explain the observed 3 to 5-fold higher incidence rates of hHF in patients with a history of established ASCVD, compared to those without a history of established ASCVD, in our study. Since there was no significant difference in hHF between dapagliflozin and empagliflozin in T2D patients with a history of established ASCVD, the selection of SGLT2 inhibitors in this population should be based on other clinical factors.

This presented study has several strengths. First, this study was based on a large multi-institutional EMR database, covering about 1.3 million individuals, to provide sufficient sample size and statistical power for the analyses. Second, many clinically relevant data related to the disease severity were considered in this study, such as laboratory parameters for patients’ baseline blood glucose levels, renal functions and blood pressure. However, several limitations should be noted due to the nature of the observational study design. First, although the diagnosis codes for heart failure have been validated in our dataset [20], possible misclassification bias cannot be ignored. We conducted two sensitivity analyses applying narrower criteria for case ascertainments, including diagnosis by cardiologists only, and the use of BNP-level data to define heart failure [38], whereby the results were consistent with the main analyses. Second, although we used propensity score methods to minimize the differences in baseline covariates between empagliflozin and dapagliflozin, some unmeasured confounders remained unresolved [57]. However, we considered the new user design with active comparator approach should minimize these biases since the impact of unmeasured confounders should be non-differential between the two treatment groups [58, 59]. For example, the study did not consider patients’ lifestyle modifications after SGLT2 inhibitor initiation. We assumed that lifestyle changes were similar between the two SGLT2 inhibitor groups, and that any differences would be of no influence. Furthermore, while the smoking status is not routinely recorded in CGRD, those demographics (e.g., age and sex) and medical disorders (e.g., COPD) associated with smoking behaviors were similar for the two SGLT2 inhibitor groups [60]. To address potential effects from unmeasured confounders, we applied the E-value approach [40, 61], and found it unlikely that an unmeasured confounder having a substantial association with the prescribing choice between dapagliflozin and empagliflozin would have a relative risk exceeding 2.35. Third, the study may be subject to incomplete follow-up data as a result of patients being transferred to other hospitals. To evaluate the effects due to loss of follow-up, we conducted on-treatment analyses, whereby the findings were consistent with the main analyses. Finally, we may have failed to capture conditions with mild to moderate symptoms of HF without hospitalization.

## Conclusion

This real-world study reported the difference in hHF risk after treatment with dapagliflozin or empagliflozin, between those with and those without a history of established ASCVD. Compared to empagliflozin, the use of dapagliflozin was associated with a lower risk of hHF in patients without established ASCVD, while this association was not observed in patients with established ASCVD. This study lays the groundwork for future prospective study to support our findings.

## Supplementary Information

**Additional file 1: Figure S1.** Overview of study design. **Figure S2.** Study patient cohort assembly flowchart. **Table S1.** Diagnosis codes for study outcome and co-morbidity. **Table S2.** Individual drug for study co-medication. **Table S3.** Baseline characteristics before 1:1 propensity score matching (original cohort). **Table S4.** Results from the Cox regression model after 1:1 propensity score matching **Table S5.** The number of patients at risk, patients with hHF outcomes, and patients censored.

## Data Availability

Data sharing is not applicable to this study as data management and analysis were performed on a statistics server through remote access in Chang Gung Medical Foundation in Taiwan, out of privacy and safety concerns.

## Abbreviations

ASCVD: Atherosclerotic cardiovascular disease
ASMD: Absolute standardized mean differences
BNP: B-type Natriuretic Peptide
CCI: Charlson comorbidity index
CI: Confidence intervals
eGFR: Estimated glomerular filtration rate
EMR: Electronic medical records
HbA1c: Glycated hemoglobin A1c
hHF: Hospitalization for heart failure
HR: Hazard ratios
ICD-9-CM: International Classification of Diseases, Ninth Revision, Clinical Modification
ICD-10-CM: International Classification of Diseases, Tenth Revision, Clinical Modification
RCTs: Randomized controlled trials
SBP: Systolic blood pressure
SD: Standard deviation
SGLT2: Sodium-glucose co-transporter 2
T2D: Type 2 diabetes
