# Supplementary material for: Differences in outcomes of hospitalizations for heart failure after SGLT2 inhibitor treatment: effect modification by atherosclerotic cardiovascular disease
Source: Cardiovasc Diabetol. 2021 Oct 23;20:213. doi: 10.1186/s12933-021-01406-3 (PMC8542324; doi:10.1186/s12933-021-01406-3)
Supplement: Supplementary file 1 — Additional file 1: Figure S1. Overview of study design. Figure S2. Study patient cohort assembly flowchart. Table S1. Diagnosis codes for study outcome and co-morbidity. Table S2. Individual drug for study co-medication. Table S3. Baseline characteristics before 1:1 propensity score matching (original cohort). Table S4. Results from the Cox regression model after 1:1 propensity score matching Table S5. The number of patients at risk, patients with hHF outcomes, and patients censored. [file 12933_2021_1406_MOESM1_ESM.pdf]

Additional File 1: Supplementary information

Figure S1. Overview of study design
Figure S2. Study patient cohort assembly flowchart
Table S1. Diagnosis codes for study outcome and co-morbidity

Table S2. Individual drug for study co-medication

Table S3. Baseline characteristics before 1:1 propensity score matching (original cohort)

Table S4. Results from the Cox regression model after 1:1 propensity score matching

Table S5. The number of patients at risk, patients with hHF outcomes, and patients censored

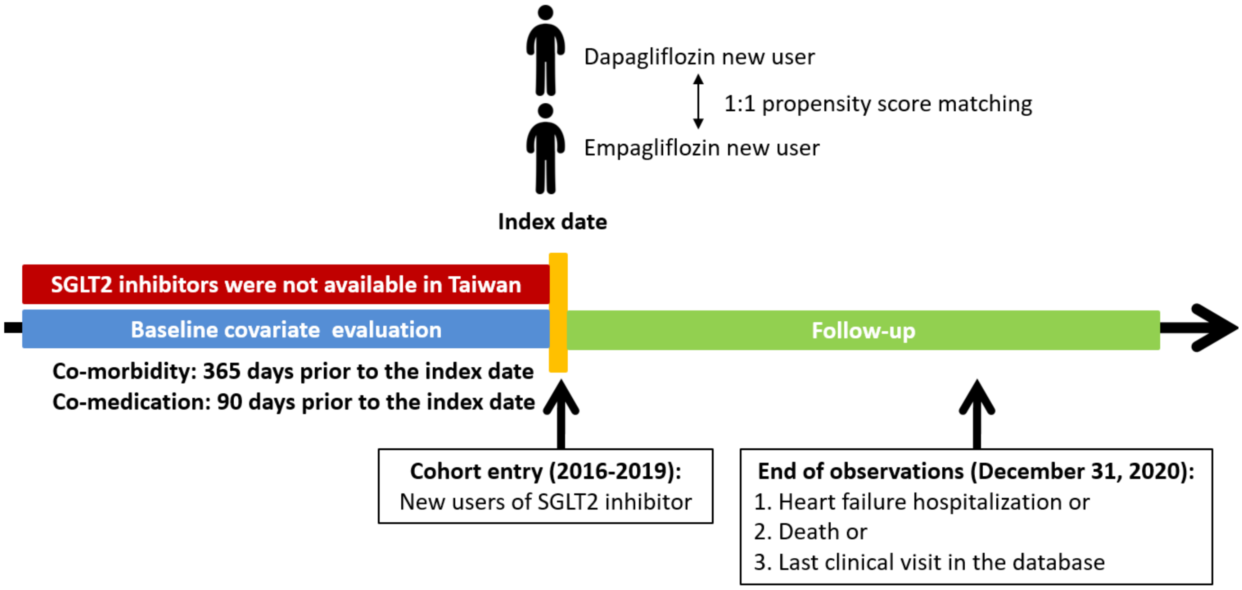

Figure S1. Overview of study design.

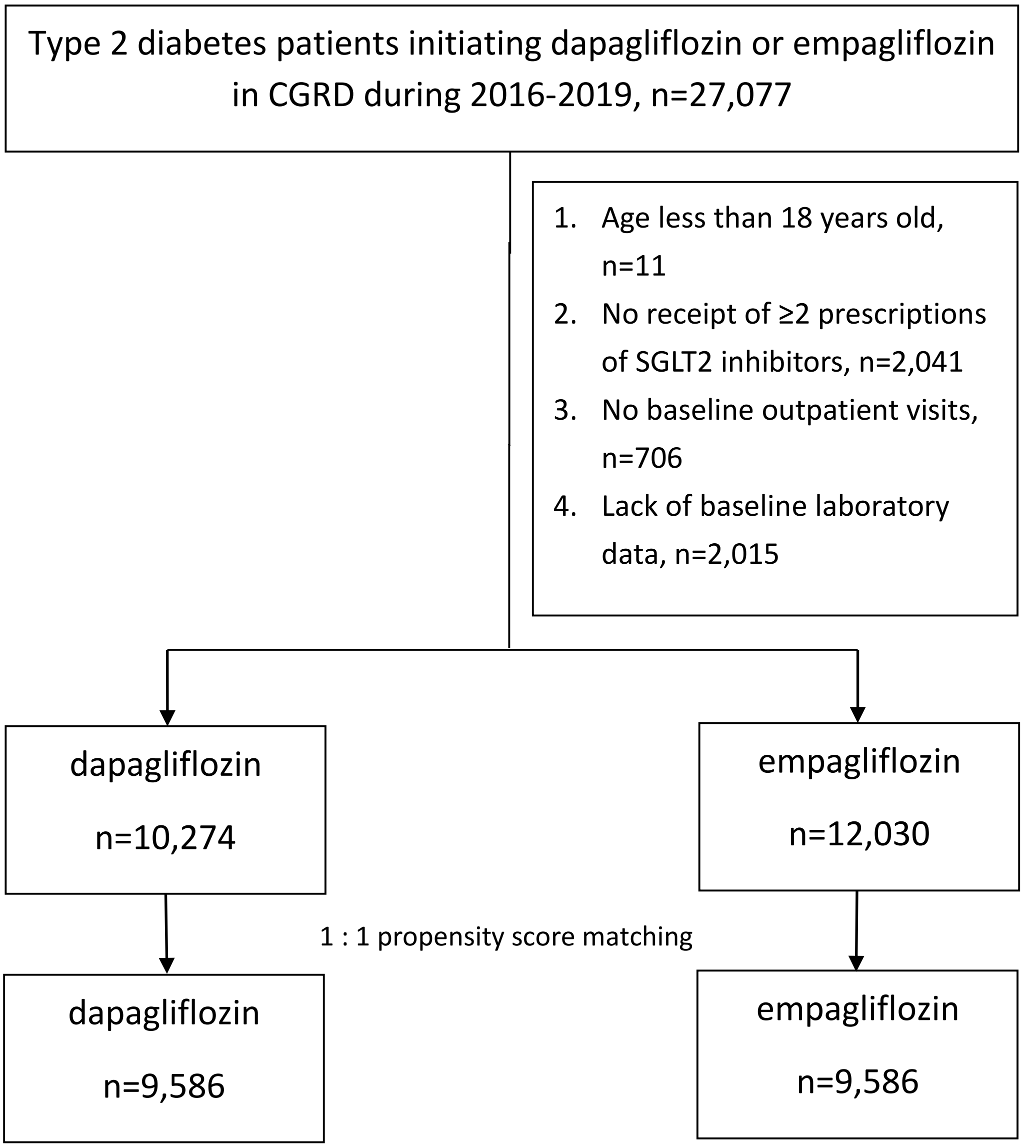

Figure S2. Study Patient Cohort Assembly Flowchart
CGRD, Chang Gung Research Database; SGLT2, sodium glucose co-transporter 2

Table S1. ICD codes of co-morbidities

Diseases	ICD-9 codes	ICD-10 codes
Hypertension	401, 402, 403, 404, 405	I10, I11, I12, I13, I15, I16
Hyperlipidemia	272	E78
ASCVD		
Coronary heart disease	410, 411, 412, 413, 414	I20, I21, I22, I23, I24, I25
Ischemic stroke	433,434	I63
Peripheral artery disease	440	I70, I73
Heart failure	428	I50
Atrial fibrillation		
Diabetic retinopathy	2505	E083, E113
Diabetic neuropathy	2506	E084, E114
Diabetic nephropathy	2504	E082, E112
COPD		
Liver disease	5712, 5715	K702, K7030, K7031, K741, K742, K7460, K7469
Depression	2962, 2963	F32, F33
Schizophrenia	295	F20
Cancer	140-239	C0-D4

Table S2. List of co-medications

Drug class	Drug name
Anti-platelet agents	aspirin, clopidogrel, dipyridamole, ticagrelor
Anti-coagulant agents	apixaban, diabagatran, edoxaban, rivaroxaban, warfarin
Beta-blocker	atenolol, bisoprolol, carvedilol, metoprolol, propranolol
Angiotensin-converting enzyme inhibitors or angiotensin receptor blockers	Azilsartan, candestartan, captopril, enalapril, fosinopril, irbesartan, losartan, olmesartan, ramipril telmisartan, valsartan
Calcium channel blockers	amlodipine, diltiazem, felodipine, lercanidipine, nifedipine, verapamil
Spironolactone	spironolactone
Other diuretics	acetazolamide, amiloride, benzyl hydrochlorothiazide, bumetanide, furosemide, Hydrochlorothiazide, Indapamide
Digoxin	digoxin
Nitrate	hydralazine, isosorbide dinitrate , isosorbide mononitrate
Statin	atorvastatin, fluvastatin, pitavastatin, rosuvastatin, simvastatin
Fibrate	fenofibrate, gemfibrozil
Ezetimibe	ezetimibe
Metformin	metformin
Sulfonylurea	glipizide, gliclazide, glimepiride, glyburide
DPP4 inhibitors	alogliptin, linagliptin, sitagliptin, vidagliptin, saxagliptin
Acarbose	acarbose
Glinides	nateglinide, meglitinide, repaglinde
Thiazolidinediones	pioglitzone
Glucagon-like peptide-1 receptor antagonists	dulaglutide, exenatide, liraglutide
Insulin	rapid, short, intermediate and long-acting insulins
NSAID	acemetacin, celecoxib, diclofenac, etodolac, etoricoxib, ibuprofen, indomethacin, meloxicam, naproxen, sulindac

Table S3. Baseline characteristics before 1:1 propensity score matching (original cohort)

	All patients			Patients with ASCVD			Patients without ASCVD		
	Dapagliflozin (N=10,274)	Empagliflozin (N=12,030)	ASMD	Dapagliflozin (N=2,447)	Empagliflozin (N=3,551)	ASMD	Dapagliflozin (N=7,827)	Empagliflozin (N=8,479)	ASMD
Age, mean (SD) years	59.5 (11.6)	60.6 (12.0)	0.09	63.5 (10.3)	64.1 (10.7)	0.05	58.3 (11.7)	59.1 (12.2)	0.07
Female, %	40.4	38.8	0.10*	28.2	26.1	0.13*	44.3	44.0	0.07
HbA1c, mean (SD) %	8.7 (1.6)	8.7 (1.8)	0.02	8.6 (1.6)	8.5 (1.7)	0.06	8.8 (1.6)	8.8 (1.8)	<0.01
SBP, mean (SD) mmHg	140.3 (19.4)	138.8 (20.0)	0.08	138.8 (20.0)	136.3 (20.5)	0.13*	140.8 (19.1)	139.9 (19.7)	0.05
DBP, mean (SD) mmHg	78.9 (12.1)	78.0 (12.1)	0.08	77.9 (12.1)	76.2 (12.2)	0.14*	79.2 (12.1)	78.8 (12.0)	0.04
eGFR, mean (SD) ml/min/1.73m^2	94.1 (30.3)	90.0 (33.1)	0.13*	86.8 (26.6)	82.3 (27.1)	0.17*	96.3 (31.0)	93.2 (34.8)	0.09
Hospital level, %			0.11*			0.09			0.14*
Medical centers	52.7	56.9		54.6	59.4		52.2	55.8	
Regional hospitals	29.5	24.1		30.2	25.4		29.3	23.6	
District hospitals	17.8	19.0		15.2	15.2		18.6	20.6	
Specialty of prescriber, %			0.16*			0.07			0.17*
Metabolism & Endocrinology	60.4	52.4		33.7	30.6		68.7	61.5	
Cardiology	27.3	32.1		55.1	57.4		18.6	21.6	
Others	12.3	15.5		11.2	12.0		12.7	17.0	
Previous hospitalizations, %	14.5	19.4	0.13*	28.4	37.1	0.19*	10.1	12.1	0.06
ASCVD, %	23.8	29.5	0.12*	100.0	100.0	-			
Coronary heart disease	20.3	25.5	0.12*	85.1	86.4	0.04	-	-	-
Ischemic stroke	3.6	3.9	0.01	15.3	13.2	0.06	-	-	-
Peripheral artery disease	1.2	1.7	0.04	5.0	5.8	0.04	-	-	-
Comorbidity, %									
Hypertension	67.0	68.7	0.04	76.7	76.0	0.02	63.9	65.6	0.03
Hyperlipidemia	73.4	69.7	0.08	72.6	68.7	0.09	73.7	70.1	0.08
Heart failure	5.3	8.9	0.14*	14.3	19.7	0.15*	2.5	4.4	0.10*
Atrial fibrillation	3.0	4.2	0.06	5.3	6.1	0.04	2.3	3.4	0.07
Diabetic retinopathy	8.2	7.5	0.03	6.7	6.3	0.01	8.7	7.9	0.03
Diabetic neuropathy	8.8	8.2	0.02	7.6	7.0	0.02	9.2	8.7	0.02
Diabetic nephropathy	23.4	24.1	0.02	16.6	19.2	0.07	25.5	26.2	0.01
COPD	3.0	3.5	0.03	5.2	5.6	0.02	2.3	2.7	0.02
Liver disease	17.4	16.9	0.01	12.1	11.5	0.02	19.1	19.2	<0.01

Depression	1.6	1.6	<0.01	1.4	1.8	0.03	1.6	1.5	0.01
Schizophrenia	0.4	0.4	<0.01	0.3	0.2	<0.01	0.5	0.5	<0.01
Cancer	6.0	6.0	<0.01	5.3	5.8	0.02	6.3	6.1	<0.01
CCI, mean (SD) scores	2.4 (1.5)	2.6 (1.6)	0.10*	2.7 (1.7)	3.0 (1.8)	0.13*	2.4 (1.4)	2.5 (1.5)	0.07
Co-medications, %									
Anti-platelet agents	31.1	37.4	0.13*	77.2	81.8	0.11*	16.7	18.9	0.06
Anti-coagulant agents	3.1	4.2	0.06	5.3	6.1	0.03	2.4	3.4	0.06
Beta blockers	27.3	31.4	0.09	54.9	57.2	0.05	18.7	20.6	0.05
ACEI / ARB	58.9	64.0	0.10*	74.1	75.7	0.04	54.2	59.0	0.10
Calcium channel blockers	39.2	41.8	0.05	50.5	44.6	0.12*	35.7	40.6	0.10*
Spironolactone	2.3	4.5	0.12*	4.5	7.9	0.14*	1.7	3.1	0.09
Other diuretics	8.7	12.0	0.11*	13.6	17.0	0.09	7.1	9.8	0.10
Digoxin	1.1	1.5	0.04	2.1	2.1	<0.01	0.7	1.2	0.05
Nitrate	6.0	8.3	0.09	19.4	21.5	0.05	1.7	2.7	0.06
Statin	65.3	68.1	0.06	76.7	80.9	0.10*	61.8	62.7	0.02
Fibrate	9.2	9.4	<0.01	8.3	7.9	0.01	9.5	10.0	0.02
Ezetimibe	11.5	12.0	0.01	14.6	15.8	0.03	10.5	10.4	<0.01
Metformin	88.6	84.9	0.11*	87.1	82.2	0.13*	89.1	86.0	0.09
Sulfonylurea	56.7	51.2	0.11*	55.7	49.1	0.13*	57.0	52.1	0.10
DPP4 inhibitors	55.9	56.5	0.01	56.5	53.7	0.06	55.6	57.8	0.04
Acarbose	15.7	15.9	<0.01	18.0	16.1	0.05	15.0	15.8	0.02
Glinides	1.8	2.2	0.03	1.4	2.2	0.06	1.9	2.2	0.03
Thiazolidinediones	23.4	19.9	0.09	20.8	18.1	0.07	24.3	20.6	0.09
GLP-1 RAs	0.9	1.3	0.04	0.7	1.0	0.03	0.9	1.4	0.04
Insulins	15.9	18.7	0.08	13.8	17.1	0.09	16.5	19.4	0.08
NSAID	7.9	8.2	0.01	8.2	7.9	0.01	7.7	8.3	0.02

*ASMD >0.1 indicates a difference between the two groups.

ACEI, angiotensin converting enzyme inhibitors; ARB, angiotensin II receptor blockers; ASCVD, atherosclerotic cardiovascular disease; ASMD, absolute standardized mean difference; CCI, Charlson Comorbidity Index; COPD, chronic obstructive pulmonary disease; DBP, diastolic blood pressure; DPP4, dipeptidyl peptidase-4; eGFR, estimated glomerular filtration rate; GLP-1 RAs, glucagon-like peptide-1 receptor agonists; HbA1c, glycated hemoglobin A1c; NSAID, non-steroidal anti-inflammatory drug; SBP, systolic blood pressure; SD, standard deviation

**Table S4.** Results from the Cox regression model after 1:1 propensity score matching					
	**Patients**	**Events**	**Follow-up (person-years)**	**Incidence rate (per 1,000 person-year)**	**HR (95% CI)**
**Overall**					
Dapagliflozin	9586	193	25528.09	7.56	0.90 (0.74-1.09)
Empagliflozin	9586	217	25869.59	8.39	Reference
**Patients with ASCVD**					
Dapagliflozin	2401	123	6194.14	19.86	1.12 (0.87-1.45)
Empagliflozin	2434	111	6300.60	17.62	Reference
**Patients without ASCVD**					
Dapagliflozin	7185	70	19333.95	3.62	0.67 (0.49-0.90)
Empagliflozin	7152	106	19568.99	5.42	Reference

**Table S5.** The number of patients at risk, patients with hHF outcomes, and patients censored.						
All patients	study entry	1 year	2 years	3 years	4 years	5 years
Patients at risk						
Emapgliflozin	9586	8728	6211	4314	2059	0
Dapagliflozin	9586	8725	5974	4109	2061	0
Patients with hHF outcomes						
Emapgliflozin	0	94	147	191	211	217
Dapagliflozin	0	86	137	169	184	193
Patients censored						
Emapgliflozin	-	764	3228	5081	7316	9369
Dapagliflozin	-	775	3475	5308	7341	9393
ASCVD patients at risk						
Patients at risk	2434	2191	1530	971	434	0
Emapgliflozin	2401	2165	1437	960	457	0
Dapagliflozin						
Patients with hHF outcomes	0	51	73	102	111	111
Emapgliflozin	0	58	91	108	117	123
Dapagliflozin						
Patients censored						
Emapgliflozin	-	192	831	1361	1889	2323
Dapagliflozin	-	178	873	1333	1827	2278
Non-ASCVD patients at risk						
Patients at risk						
Emapgliflozin	7152	6537	4681	3343	1625	0
Dapagliflozin	7185	6560	4537	3149	1604	0
Patients with hHF outcomes						
Emapgliflozin	0	43	74	89	100	106
Dapagliflozin	0	28	46	61	67	70
Patients censored						
Emapgliflozin	-	572	2397	3720	5427	7046
Dapagliflozin	-	597	2602	3975	5514	7115
